
JOURNAL INFORMATION
==============================
NLM Title Abbreviation: Gastroenterol Rep (Oxf)
Journal ID: Gastroenterol Rep (Oxf)
NLM Journal ID: 101620508
Journal ID: gastro

Gastroenterology Report

EISSN: 2052-0034
Publisher: Oxford University Press

ARTICLE INFORMATION
==============================
PMCID: PMC4650982
DOI: 10.1093/gastro/gov060
Article ID: gov060
Article version: 1
Subjects: Chinese Abstracts

Chinese Abstracts – Volume 3 Issue 4*

*This article abstract was originally published in English. This translation has not been verified and should not be relied upon—it is provided for reference purposes only. The Publishers have not checked this translation and accept no liability for completeness or accuracy of this translation or the use of this translation for whatever purpose. This translation may be incomplete and inaccurate in whole or in part. If you need to rely upon a translation of this abstract, a professional human translator should be engaged to supply an accurate translation of the original English. When referencing articles from this journal, please always refer to the original English version, rather than a translated equivalent.

Print publication date: 2015 Nov
Electronic publication date: 2015 Nov 16
Volume: 3
Issue: 4
Issue title: Special Issue: Screening and Comprehensive Treatment of GI Tumours
First page: C1
Last page: C2
Copyright: © The Author(s) 2015. Published by Oxford University Press and the Digestive Science Publishing Co. Limited.
Copyright year: 2015
License: This is an Open Access article distributed under the terms of the Creative Commons Attribution License (http://creativecommons.org/licenses/by/4.0/), which permits unrestricted reuse, distribution, and reproduction in any medium, provided the original work is properly cited.
License URL: https://creativecommons.org/licenses/by/4.0/

Page count: 2

==============================
OXFORD

*Gastroenterology Report*, 2015, 1–2

doi: 10.1093/gastro/gov060

### Chinese Abstracts – Volume 3 Issue 4*

### Review（综述）

### Molecular phenotypes of colorectal cancer and potential clinical applications
### 结直肠癌分子分型及其潜在临床意义

[摘要] 结直肠癌是一种具有多种可能发生途径的异质性疾病。这种异质性对于结直肠癌的预后和临床治疗具有非常重要的意义。因此，结直肠癌发生风险和预后的流行病学研究，以及治疗相关的临床试验，对于结直肠癌分子分型这个问题都非常重视。本文描述了可反映结直肠癌异质性且受到广泛研究的4种肿瘤标记物：微卫星不稳定性（MSI）或DNA错配修复（MMR）缺失、CpG岛甲基化表型（CIMP）、*BRAF*突变及*KRAS*突变。这些肿瘤标记物已被证实可以更好地定义结直肠癌的流行病学特征，而且也越来越多地应用于指导临床治疗决策。除了这些传统的肿瘤标记物外，我们还简述了一些具有潜在临床价值的新型标记物。最终，我们应认清，所有的肿瘤标记物并不是单独作用，而是可以通过有机组合成为肿瘤分子分型。本文综述了几种结直肠癌病因学通道假说的研究进展，并对其各自的临床意义进行了深入探讨。

[关键词] 结直肠癌；微卫星不稳定；CpG岛甲基化表型；KRAS；BRAF；治疗；流行病学

### Neoadjuvant chemotherapy and pathological complete response in rectal cancer
### 直肠癌新辅助放化疗和病理完全缓解

[摘要] 在过去几十年里，直肠癌的治疗已有了很大的进展，其中最为显著的要属20世纪90年代全直肠系膜切除术（TME）的问世和新辅助放化疗（CRT）的应用。临床Ⅰ级证据显示，新辅助放化疗（CRT）可使局部复发率降至6%以下，CRT也就此成为了目前直肠癌的标准治疗模式。CRT 可使肿瘤降期，其中15%－27%的患者可以获得病理完全缓解（pCR），意味着CRT后在组织学标本中无任何肿瘤残留。CRT 后获得pCR 的患者预后良好，局部复发和远处转移风险下降，生存时间延长。因此，积极保留括约肌或保留器官的手术方式如局部切除术在直肠癌治疗中得以更多的应用，甚至非手术治疗也成为可能。尽管多学科综合治疗可使局部进展期直肠癌的局部复发率稳定在5%－6%，但其远处转移率却仍然高达25%以上，这也是患者的主要死因。因此，最近一些研究采用新辅助全量系统化疗以早期治疗远处微转移灶，从而提高肿瘤的系统控制率，仅对化疗无应答者或低位直肠癌（距肛缘不足5 cm）进行术前放疗。

[关键词] 直肠癌；新辅助放化疗；病理完全缓解

### Immune checkpoints and immunotherapy for colorectal cancer
### 结直肠癌的免疫调控点和免疫治疗

[摘要] 尽管过去10年间结直肠癌在早期诊断水平和总体生存改善方面有着稳步提高，但其仍是世界范围内主要致死疾病之一。目前包括化疗和生物治疗在内的治疗模式似乎已经达到了一个很高的高度，然而，免疫治疗尤其是免疫调控点抑制剂，也已在包括错配修复缺失的结直肠癌在内的多种癌症治疗中显示出良好的临床效果。由此萌发出一些针对免疫治疗新药进行评估（包括单药、多药、与化疗联合应用）的临床试验的设计与实施。本文对这些免疫调控抑制剂的生物学特性和临床前数据进行了综述，并对多项结直肠癌免疫治疗相关的临床试验和目前结直肠癌免疫治疗的努力方向进行了讨论。

[关键词] 免疫治疗；结直肠癌；免疫调控点抑制/阻断；程序性死亡因子1（PD-1）；T淋巴细胞相关抗原4（CTLA-4）；疫苗；Pembrolizumab

### Treatment of peritoneal metastases from colorectal cancer
### 结直肠癌腹膜转移的治疗

[摘要] 腹膜种植是结直肠癌第2常见的转移部位（仅次于肝转移）。在5-FU单药化疗时代，结直肠癌腹膜转移的预后非常差。近年来，尽管一些新的化疗和靶向治疗药物的应用已使腹膜转移的预后得以改善，但其治疗反应似乎仍然不及肝转移。减瘤手术和腹腔热灌注化疗联合应用已被证实可以进一步提高腹膜转移患者的无瘤生存及总体生存时间。因此，每一位结直肠癌腹膜转移患者都应接受个体化综合评估，如果可能的话，应予以充分的联合治疗。本文综述了腹膜转移治疗方面的最新研究进展。

[关键词] 结直肠癌；腹膜转移；减瘤手术；腹腔化疗

* This article abstract was originally published in English. This translation has not been verified and should not be relied upon—it is provided for reference purposes only. The Publishers have not checked this translation and accept no liability for completeness or accuracy of this translation or the use of this translation for whatever purpose. This translation may be incomplete and inaccurate in whole or in part. If you need to rely upon a translation of this abstract, a professional human translator should be engaged to supply an accurate translation of the original English. When referencing articles from this journal, please always refer to the original English version, rather than a translated equivalent.
本文摘要最初是以英文发表。中文摘译未获认证，谨供参考。出版社没有对中文摘译进行核验，不负责译文的完整性和准确性，也不会将其用以其他目的。中文摘译可能会出现部分不完整或不准确的情况。如果你需要准确获悉摘要，最好能请一位专业译者针对英文原文进行翻译。阅读杂志文章，请参考英文原文，不推荐中文翻译。

### Advances in the management of Barrett's esophagus and esophageal adenocarcinoma
### Barrett食管和早期食管腺癌的治疗进展

[摘要] 在过去几十年间，美国早期食管癌的发生率快速增长。Barrett食管是癌变最为显著的危险因素。理论上，Barrett 食管的早期筛查和早期治疗有助于早期食管癌的预防，然而，Barrett食管及其恶性转化的发生率仍不甚清楚，而且也缺少 Barrett 食管筛查的成本－效应研究结果。过去几十年间，我们对Barrett食管流行病学特征、病理发生机制及内镜治疗方面的认识有了一些重大进展。这些进展主要集中在组织学进展的早期识别和高级别上皮内瘤变的内镜治疗。内镜切除技术的进步已使我们能够在内镜下治疗早期食管癌。本文将详细探讨Barrett食管和早期食管腺癌的诊治进展。

[关键词] Barrett食管；早期食管腺癌；内镜

### Targeted therapy in gastroesophageal cancers: past, present, and future
### 胃/食管癌的靶向治疗：过去、现在和将来

[摘要]　　胃/食管癌是一个全球难题，发现时经常已处于无法根治的阶段，对其进行标准方案化疗，预后也非常差。本文首先总结胃/食管癌的流行病学及分子生物学特征，然后重点阐述靶向治疗相关的几条关键信号调控通路。同时，本文还对靶向药物的临床应用情况（包括成功与失败的数据）进行了全面回顾。值得一提的是，靶向HER2的单克隆抗体 trastuzumab 在一线治疗中的获益已获证实，从而作为一项标准治疗应用于临床实践。靶向 VEGF 的单克隆抗体 ramucirumab 的疗效也获证实，并被批准用于临床二线治疗。相比这些积极的研究结果，虽然 EGFR 和 MET 通路的靶向治疗药物在胃/食管癌中也有着广泛的研究，但其疗效并未获得证实。有必要对预测这些靶向药物疗效的分子标记物进行更深入的研究。未来，基于抑制上述信号通路的相关研究结果而出现的更为可靠的分子病理学方法，或可将胃/食管癌这种异质性疾病进行亚分类，从而实现个体化治疗。精准的病例选择、合理的靶向药物搭配以及免疫治疗药物的联合应用有望进一步提高胃/食管癌的疗效。

[关键词] 食管癌；胃癌。靶向治疗；分子肿瘤

### Advances in the prevention and management of gastrointestinal cancers. Endoscopic resection of gastric and esophageal cancer
### 胃肠道肿瘤的预防和治疗进展：胃癌和食管癌的内镜治疗

[摘要] 内镜黏膜下剥离术（ESD）和内镜黏膜切除术（EMR）可以减少早期食管癌和早期胃癌对手术治疗的需求，从而降低了其并发症发生率和病死率。ESD 在西方国家是一个相对较新的技术，需要严格的技术培训以达到韩国、日本等亚洲国家的技术熟练度，在这些国家，ESD 可以获得非常高的完整切除率，且并发症发生率非常低。EMR 技术在早期食管癌中具有重要意义。相比之下，ESD 的完整切除率更高，而 EMR 更容易掌握且操作时间更短。对于由 Barrett 食管引起的早期食管腺癌，ESD 和 EMR 技术常与其他消融技术（最常用的是射频消融）联合应用，已有大量数据显示其高成功率。虽然 EMR 技术应用于早期胃癌也取得了一些成功，但目前在早期胃癌中，ESD的应用更为普遍。ESD 可切除至黏膜下层，从而可以对早期胃癌进行更为积极（更深层）的内镜切除。

[关键词]　内镜黏膜下剥离术；内镜黏膜切除术；内镜消融；早期胃癌；早期食管癌

### Original article（论著）

### Survival of patients with KRAS wild-type metastatic colorectal cancer is identical after sequential treatment with cetuximab and bevacizumab regardless of the sequenceĐA retrospective single center study
### 西妥昔单抗和贝伐单抗序贯治疗用于KRAS野生型转移性结直肠癌，其治疗先后顺序不会影响患者生存时间：一项回顾性单中心研究

[摘要]　目的：研究KRAS野生型转移性结直肠癌患者在接受西妥昔单抗和贝伐单抗序贯治疗后的总体生存情况。方法：回顾性分析在中山大学附属胃肠肛门医院接受西妥昔单抗和贝伐单抗序贯治疗的26例转移性结直肠癌病例，分为A、B两组，A组病例先予以贝伐单抗，B组病例先予以西妥昔单抗。比较两组患者治疗反应率、无进展生存时间和总生存时间。结果：两组病例基线资料相似。A、B两组患者客观反应率分别为62.5%和66.6%（$P$=0.132），中位无进展生存时间分别为13月和10月（$P$=0.798）。全组病例中位总生存时间为42月，其中A组44月，B组39月，两组差异无统计学意义（$P$=0.862）。年龄<40岁者总生存时间较≥40岁者明显缩短（中位时间：22 *vs* 44月，$P$=0.029）。同时性转移者较异时性转移者预后更差（前者未达到中位时间，后者中位总生存时间为36月）。多因素分析显示，同时性转移（HR=4.548）和年龄≥40岁（HR=0.237）是本组病例的独立预后因素。结论：无论顺序为何，靶向序贯治疗用于转移性结直肠癌可使患者有一个较长的的生存获益，值得进一步研究。

[关键词] 转移性结直肠癌；序贯治疗；贝伐单抗；西妥昔单抗

### p53 overexpression predicts colorectal neoplasia risk in patients with inflammatory bowel disease and mucosa changes indefinite for dysplasia
### p53过表达可预测具有不典型增生不确定型黏膜改变的炎性肠病患者的瘤变风险

[摘要] 背景和目的：我们之前研究发现，具有不典型增生不确定型黏膜改变（IND）的炎性肠病（IBD）患者具有较高的瘤变风险，同时发现，p53和细胞角蛋白7免疫组化检测对于IBD相关的瘤变具有潜在的诊断价值。本研究旨在初步论证这两种标记物对于 IBD-IND 患者瘤变风险的预测价值。方法：我们从病理数据库中筛选出了44例符合研究纳入标准且经组织学证实的IBD-IND患者。采用免疫组化方法对所有结肠标本进行p53和细胞角蛋白7的半定量检测，分析这两种标记物的表达、人口统计学特征及临床资料与结直肠瘤变的相关性。结果：本组病例平均年龄（46.6 – 15.1）岁，男性患者25例（56.8%）。在IND诊断后，中位随访101（6 – 247）月。11例（25%）患者在随访期间发生瘤变，其中低级别上皮内瘤变6例，高级别上皮内瘤变3例，癌变3例。这 11 例患者的中位随访时间为66（19 – 145）月。单因素分析显示，年龄和 p53 过表达与瘤变相关。结论：有25%的IBD-IND患者会进展为不典型增生或癌。p53过表达和年龄与IND-IBD的瘤变相关。

[关键词] 炎性肠病；溃疡性结肠炎；克罗恩病；结直肠瘤变；p53；细胞角蛋白7

### Case report（病例报告）

### Management of sub-5 mm rectal carcinoids with lymph node metastases
### 合并淋巴结转移的微小直肠类癌（<5 mm）的处理
